# Heterogeneity in preferences for HIV prevention service delivery among women of reproductive age in western Kenya: a latent class analysis

**DOI:** 10.3389/fgwh.2026.1810230

**Published:** 2026-06-25

**Authors:** Melissa Latigo Mugambi, Ben O. Odhiambo, Annabell Dollah, Mary M. Marwa, Judith Nyakina, John Kinuthia, Grace John-Stewart, Ruanne Vanessa Barnabas, Brett Hauber

**Affiliations:** 1 Department of Global Health, University of Washington, Seattle, WA, United States; 2 Department of Research and Programs, Kenyatta National Hospital, Nairobi, Kenya; 3 UW-Kenya, Nairobi, Kenya; 4 Department of Medicine, University of Washington, Seattle, WA, United States; 5 Department of Epidemiology, University of Washington, Seattle, WA, United States; 6 Department of Pediatrics, University of Washington, Seattle, WA, United States; 7 Division of Infectious Diseases, Massachusetts General Hospital and Harvard Medical School, Boston, MA, United States; 8 The Comparative Health Outcomes, Policy and Economics (CHOICE) Institute, Department of Pharmacy, University of Washington, Seattle, WA, United States; 9 Pfizer, Inc, New York, NY, United States

**Keywords:** HIV infections, Kenya, patient preference, pregnancy, prevention & control, women

## Abstract

**Introduction:**

Pharmacies might create opportunities for pregnant women to initiate HIV prevention services earlier and more regularly, but their role in HIV prevention during pregnancy—a high-risk period for HIV acquisition—remains relatively unexplored. We evaluated whether women of childbearing age have different preferences for accessing HIV prevention services in Western Kenya.

**Methods:**

We administered a discrete choice experiment (DCE) survey to women aged 15–44 in Western Kenya from June to November 2023. The survey asked women to choose among HIV prevention services described by seven attributes: service location, travel time, type of HIV test, sexually transmitted infection (STI) testing, partner HIV testing, pre-exposure prophylaxis (PrEP), and service fee. We used a latent-class model with effects-coded attribute levels to explore preference heterogeneity.

**Results:**

Among the 599 women included in the final analysis, the median age was 23 years (IQR: 18–27). 213 (33%) were married or living with a partner, and 311 (52%) had a prior pregnancy. The latent class analysis identified four distinct groups: the clinic-seeking group (*n* = 108, 18.0%), the all-about-PrEP group (*n* = 187, 31.2%), the all-about-STI testing group (*n* = 224, 37.4%), and the price-sensitive group (*n* = 80, 13.4%).

**Conclusions:**

Women in this study exhibited heterogeneous preferences for accessing HIV prevention services. Future service design goals might prioritize STI testing and PrEP implementation, ensure targeted improvements to clinic-based services for women who value this setting, and offer flexible pricing to accommodate price-sensitive individuals.

## Introduction

In western Kenya, a high–HIV–burden setting (specifically Homa Bay, Siaya, and Kisumu counties), the median gestational age at which women initiate antenatal care is 4.8–4.9 months, while roughly 63%–69% of women attend at least four antenatal care visits (1), limiting timely access to beneficial preventive HIV services. Over the past decade, researchers and implementers have explored alternative service delivery models to ensure that pregnant women initiate antenatal care early and consistently in Kenya and across the broader Eastern and Southern African regions (2–4). These models have included community health workers and pharmacies and aim to address human resource constraints through task shifting and by creating more touchpoints for accessing services. Pharmacies have also been explored as an alternative service model through which adolescent girls and young women can access HIV prevention services, and they are increasingly recognized as an important touchpoint (5). Given the growing interest in engaging pharmacies, understanding how to serve pregnant populations is important. However, research on women's preferences for delivery of HIV prevention services during pregnancy is limited: a study in Kenya focused on preferences for specific product attributes like long-acting PrEP (6), while another in Zambia examined PrEP delivery preferences, with the location attribute mainly focusing on facility or home options (7).

While these studies have focused on PrEP, the objective of our body of research has been to examine women's preferences for delivery of HIV prevention services, including HIV testing, PrEP, STI testing, and partner HIV testing, more broadly, and to explore whether pharmacies are important access points to women. In a prior discrete choice experiment (DCE) we conducted, 36% of women preferred accessing HIV prevention services at pharmacies with private rooms, underscoring the need for person-centered and differentiated service delivery models rather than a one-size-fits-all approach (8).

However, because pharmacy-based models should complement, rather than replace, existing antenatal care services, we need to determine whether there are groups of women with differing healthcare access needs and preferences. Therefore, we conducted a latent class analysis to identify subgroups of women with distinct preferences for HIV prevention service delivery models in the context of a hypothetical pregnancy. Understanding these heterogeneous care-seeking behaviors can enable us to design evidence-based services that cater to the diverse needs of women (9).

## Methods

We have already described how participants were recruited, how the survey was developed, and how data were collected in a prior publication (8). Briefly, we initially defined attributes and levels describing HIV prevention services based on the literature and qualitative interviews with women, health providers, and technical experts (5, 10–12). We conducted think-aloud cognitive interviews to pre-test and refine the wording of the preliminary survey instrument (13). We generated a D-efficient design in Ngene using near-zero priors that reflected the logical preference order of the attribute levels (ChoiceMetrics, version 1.3.0, 2021) (14). The design paired hypothetical service alternatives, minimized overlapping attributes, and excluded dominant alternatives. Altogether, 72 hypothetical service alternatives were generated and divided into six blocks, each containing 12 questions. We then exported the design and implemented the survey using Lighthouse Studio 9 (Sawtooth Software, version 9.15.0). The order of the choice questions was randomized within each block 50 times, generating 300 versions of the survey. Participant assignment to the survey versions proceeded sequentially through all 300 versions, then started again, so participants were evenly distributed across the six blocks. Each choice question presented participants with two hypothetical service profiles described by attribute levels, along with an opt-out option labeled “none, I don't want any HIV prevention services in pregnancy.” Participants were instructed to select the “none” option if they did not want any of the services shown. The survey included a description of the attributes and levels, along with two practice questions to prepare participants for the DCE. We conducted a pilot test with 42 women to assess survey clarity, attribute salience, and the appropriateness of the service fee range.

The final attributes and levels included: (1) the location where services are received (a clinic for pregnant women, a pharmacy with a private room, or a pharmacy without a private room); (2) travel time to the location (15, 30, or 60 min); (3) type of HIV test (self-testing with a saliva test, self-testing with a blood-based finger prick test, or a provider-administered blood-based finger prick test); (4) whether an HIV test can be taken to a partner (not available or available); (5) availability of STI testing (not available or available); (6) availability of PrEP pills (not available or available); and (7) cost of services (no cost, 400 KES, or 1,000 KES). We used the term 'clinic for pregnant women' because cognitive interviews revealed that women were more likely to use this term and were less familiar with terms such as “antenatal care clinic” or “maternal and child health clinic”. For partner testing, participants were informed that before taking a test to a partner, the provider would give instructions on how to conduct the test. For STI testing, participants were informed that sexually transmitted infections (STIs), such as Syphilis, Gonorrhea, and Chlamydia, can be transmitted during sex. STI testing would involve providing a urine sample for Gonorrhea and Chlamydia testing and having the provider conduct a blood-based finger prick test for Syphilis. Any STIs found would be treated (8).

We recruited women aged 15–44 years from prior PrEP studies, pharmacies, maternal and child health clinics, and family planning clinics (5, 11, 12, 15, 16). Women were eligible if they self-reported being HIV-negative, were aged 15–44, had ever been pregnant or planned to become pregnant within the next five years, and were willing to join the study. Women under 18 were eligible only if they had previously been pregnant, as they are considered emancipated minors (8). From June to November 2023, trained research assistants administered the survey in person. Participants answered 12 choice questions, selecting between two service alternatives or opting out (“no services”). Participants also answered questions about their sociodemographic characteristics, prior experiences with HIV prevention services, attitudes toward pharmacy and clinic-based care, and perceptions of survey difficulty.

To identify whether groups of women had different service delivery preferences, we applied a latent class model in Sawtooth Software with effects-coded attribute levels, constraining levels within each attribute to sum to zero. We tested models with 2–10 classes, using 50 estimation runs per class from different random starting points. The algorithm iterated until the improvement in log-likelihood was less than 0.001, with a maximum of 100 iterations per replication. The replication with the highest likelihood was retained for each class size. Fit statistics and the average maximum membership probabilities for the 2–6-class solutions are shown in Table 1. We chose the 4-class solution based on interpretability and fit, as it had a relatively low BIC compared with most class solutions and identified a meaningful additional class beyond the 3-class solution. We also examined how class membership varied by participant characteristics, using modal posterior assignment (assigning each respondent to the class with the highest posterior probability) to determine class membership. Table 2 shows the mean preference weight estimates, standard errors, and t-ratios for the attribute levels by latent class group. Higher mean preference weights suggest greater preference on average for that attribute level.

**Table 1:** Fit statistics and average maximum membership probability for the 2–6 class solutions.

**Groups**	**Replication**	**Log-likelihood**	**R^2^**	**Pct Cert**	**AIC**	**CAIC**	**BIC**	**Average Maximum Membership Probability**
2	25	−3,750.03	0.5251	52.51	7,550.05	7,747.05	7,722.05	0.93596
3	20	−3,652.89	0.5374	53.74	7,381.78	7,681.23	7,643.23	0.86537
4	28	−3,598.79	0.5443	54.43	7,299.58	7,701.47	7,650.47	0.85066
5	1	−3,562.52	0.5489	54.89	7,253.03	7,757.36	7,693.36	0.84275
6	21	−3,531.96	0.5527	55.27	7,217.92	7,824.69	7,747.69	0.81486

**Table 2:** Attribute level preference weights by latent class group (*N* = 599).

Label	Overall *Preference weight estimate (standard error; t-ratio)*	Group 1 (*n* = 108) *"clinic-seeking"*	Group 2 (*n* = 187) *"all-about-PrEP"*	Group 3 (*n* = 224) *"all-about-STI-testing"*	Group 4 (*n* = 80) *"price-sensitive"*
None	−3.6983 (0.1171; −31.58)	−26.8391 (not estimated)	−2.4665 (0.1425; −17.31)	−5.8846 (0.6214; −9.47)	−3.4130 (0.2727; −12.52)
Location					
Clinic	0.1544 (0.0234; 6.58)	0.2682 (0.0453; 5.92)	0.5532 (0.0882; 6.27)	0.1379 (0.0469; 2.94)	0.0542 (0.0681; 0.80)
Private Pharmacy	−0.0115 (0.0244; −0.47)	0.0125 (0.0482; 0.26)	−0.1158 (0.0803; −1.44)	0.2091 (0.0475; 4.41)	−0.3126 (0.0750; −4.17)
Pharmacy	−0.1429 (0.0248; −5.76)	−0.2807 (0.0478; −5.88)	−0.4374 (0.0875; −5.00)	−0.3471 (0.0511; −6.79)	0.2584 (0.0734; 3.52)
Travel Time					
15 min	0.0356 (0.0248; 1.44)	0.0080 (0.0493; 0.16)	0.6122 (0.0966; 6.34)	−0.0778 (0.0465; −1.68)	0.1083 (0.0716; 1.51)
30 min	0.0137 (0.0246; 0.55)	0.0354 (0.0478; 0.74)	−0.3166 (0.0831; −3.81)	−0.0169 (0.0503; −0.34)	0.0359 (0.0719; 0.50)
60 min	−0.0493 (0.0237; −2.08)	−0.0434 (0.0458; −0.95)	−0.2956 (0.0847; −3.49)	0.0947 (0.0478; 1.98)	−0.1442 (0.0690; −2.09)
HIV Test Type					
Saliva Test	−0.0036 (0.0249; −0.15)	−0.0977 (0.0493; −1.98)	0.0531 (0.0831; 0.64)	−0.1792 (0.0511; −3.50)	0.1438 (0.0773; 1.86)
Blood Test	−0.0407 (0.0249; −1.63)	−0.0985 (0.0468; −2.10)	−0.3085 (0.0923; −3.34)	0.0181 (0.0510; 0.36)	0.1445 (0.0724; 2.00)
Provider Test	0.0443 (0.0236; 1.88)	0.1962 (0.0473; 4.15)	0.2553 (0.0847; 3.01)	0.1611 (0.0455; 3.54)	−0.2883 (0.0721; −4.00)
STI Testing					
STI not available	−0.4544 (0.0170; −26.70)	−0.0937 (0.0306; −3.07)	−0.6099 (0.0624; −9.78)	−1.0450 (0.0424; −24.63)	−0.3167 (0.0476; −6.66)
STI available	0.4544 (0.0170; 26.70)	0.0937 (0.0306; 3.07)	0.6099 (0.0624; 9.78)	1.0450 (0.0424; 24.63)	0.3167 (0.0476; 6.66)
Partner HIV Testing					
Partner test not available	−0.3305 (0.0167; −19.82)	−0.1056 (0.0304; −3.47)	−0.9184 (0.0801; −11.47)	−0.6434 (0.0389; −16.55)	−0.4386 (0.0508; −8.64)
Partner test available	0.3305 (0.0167; 19.82)	0.1056 (0.0304; 3.47)	0.9184 (0.0801; 11.47)	0.6434 (0.0389; 16.55)	0.4386 (0.0508; 8.64)
PrEP Availability					
PrEP not available	−0.6146 (0.0167; −36.82)	−0.1367 (0.0300; −4.56)	−2.1127 (0.0986; −21.43)	−0.6791 (0.0355; −19.11)	−0.5621 (0.0518; −10.86)
PrEP available	0.6146 (0.0167; 36.82)	0.1367 (0.0300; 4.56)	2.1127 (0.0986; 21.43)	0.6791 (0.0355; 19.11)	0.5621 (0.0518; 10.86)
Service Fee					
Free	0.2112 (0.0239; 8.85)	0.0599 (0.0459; 1.30)	0.3131 (0.0826; 3.79)	0.1894 (0.0465; 4.07)	1.1080 (0.0827; 13.40)
400 KES	0.0501 (0.0248; 2.02)	0.0732 (0.0471; 1.56)	−0.2976 (0.0905; −3.29)	0.1083 (0.0491; 2.21)	0.0201 (0.0684; 0.29)
1,000 KES	−0.2614 (0.0240; −10.88)	−0.1331 (0.0481; −2.77)	−0.0155 (0.0747; −0.21)	−0.2976 (0.0485; −6.13)	−1.1281 (0.0819; −13.78)

Each cell shows the preference weight estimate (standard error; t-ratio). Preference weights represent relative preferences for each level within each attribute. Higher values indicate stronger preference. We were unable to estimate the standard error for the Group 1 opt-out (*N*one) parameter because 0/108 respondents in Group 1 selected the opt-out alternative across all choice tasks. Therefore, there is no within-class variation around this preference weight estimate.

### Ethical approval

This study was approved by the University of Nairobi Ethical Research Committee, the National Commission for Science, Technology, and Innovation (*N*ACOSTI), and the University of Washington Institutional Review Board. All participants provided written informed consent prior to participation.

## Results

From June to November 2023, we screened 645 women, of whom 600 were eligible and enrolled. We excluded one participant who selected the same letter option across all choice tasks (i.e., straightlining). Survey completion times were reviewed, resulting in no further exclusions. Most participants (88%) found the survey normal or easy to complete (5-point scale from very difficult to very easy). In the final analysis, we included data from 599 women. Participants were evenly distributed across three counties: Kisumu (*n* = 200, 33%), Homa Bay (*n* = 199, 33%), and Siaya (*n* = 200, 33%), with 18% (*n* = 107) having participated in prior studies. Overall, the median age was 23 years (IQR: 18–27); 33% (*n* = 213) were married or living with a partner, 20% (*n* = 118) had a job and worked regularly, and 52% (*n* = 311) had been pregnant before (Table 3). The latent class analysis identified four distinct groups of women, named according to the attribute each group considered most important: the clinic-seeking (*n* = 108, 18.0%), all-about-PrEP (*n* = 187, 31.2%), all-about-STI testing (*n* = 224, 37.4%), and price-sensitive group (*n* = 80, 13.4%) (Table 2).

**Table 3:** Participant demographics, behavioral characteristics, and attitudes toward pharmacies.

Participant characteristics and pharmacy attitudes	Total (*n* = 599)	Group 1 (clinic-seeking)	Group 2 (all-about-PrEP)	Group 3 (all-about-STI-testing)	Group 4 (price sensitive)	*p*-value
		(*n* = 108) 18%	(*n* = 187) 31%	(*n* = 224) 37%	(*n* = 80) 13%	
Age (y) Median (interquartile range)	23 (18, 27)	23 (18, 27.5)	23 (19, 27)	23 (18, 27)	20 (17, 25.5)	0.16
County location						
Kisumu	200	36 (18%)	59 (30%)	78 (39%)	27 (14%)	0.006
Homa Bay	199	22 (11%)	77 (39%)	77 (39%)	23 (12%)	
Siaya	200	50 (25%)	51 (26%)	69 (35%)	30 (15%)	
Recruitment Site						
Prior studies	107	21 (20%)	35 (33%)	37 (35%)	14 (13%)	0.763
Health facilities	193	32 (17%)	58 (30%)	71 (37%)	32 (17%)	
Pharmacies	299	55 (18%)	94 (31%)	116 (39%)	34 (11%)	
Relationship status						
Married/Living with a partner	213	45 (21%)	63 (30%)	84 (39%)	21 (10%)	0.453
Single/Divorced/Separated	207	32 (15%)	66 (32%)	77 (37%)	32 (15%)	
In a relationship	179	31 (17%)	58 (32%)	63 (35%)	27 (15%)	
Education completed						
Never attended school/At least some primary school	205	47 (23%)	58 (28%)	66 (32%)	34 (17%)	0.01
At least some secondary school	255	35 (14%)	83 (33%)	99 (39%)	38 (15%)	
At least some higher education	139	26 (19%)	46 (33%)	59 (42%)	8 (6%)	
Employment status						
Has a job and works regularly	118	18 (15%)	42 (36%)	45 (38%)	13 (11%)	0.669
Has work, but it's not regular or consistent	106	24 (15%)	28 (26%)	44 (41%)	10 (9%)	
Currently does not have a job	229	41 (18%)	71 (31%)	82 (36%)	35 (15%)	
Currently in school and not working	146	25 (17%)	46 (32%)	53 (36%)	22 (15%)	
Pharmacy visits in the past 12 months						
Once a month or more	376	61 (16%)	124 (33%)	145 (38%)	46 (12%)	0.159
Every 2 or 3 months	133	27 (20%)	41 (31%)	50 (38%)	15 (11%)	
1 or 2 times a year or not at all	90	20 (22%)	22 (24%)	29 (32%)	19 (21%)	
Travel time to regularly visited pharmacy						
Less than 15 min	311	62 (20%)	82 (26%)	126 (41%)	41 (13%)	0.166
15–29 min	202	32 (16%)	76 (38%)	65 (32%)	29 (14%)	
≥30 min	86	14 (16%)	29 (34%)	33 (38%)	10 (12%)	
Prior pregnancy						
Yes	311	57 (18%)	84 (27%)	120 (39%)	50 (16%)	0.057
No	288	51 (18%)	103 (36%)	104 (36%)	30 (10%)	
Conducted HIV test when not pregnant						
Yes	520	98 (19%)	156 (30%)	204 (39%)	62 (12%)	0.005
No	79	10 (13%)	31 (39%)	20 (25%)	18 (23%)	
Conducted STI test when not pregnant						
Yes	208	43 (21%)	68 (33%)	75 (36%)	22 (11%)	0.456
No	390	65 (17%)	118 (30%)	149 (38%)	58 (15%)	
Not sure	1	0	1 (100%)	0	0	
Took PrEP when not pregnant						
Yes	88	16 (18%)	37 (42%)	27 (31%)	8 (9%)	0.089
No	511	92 (18%)	150 (29%)	197 (39%)	72 (14%)	
Pharmacists can help you with your illness						
Agree	476	81 (17%)	147 (31%)	182 (38%)	66 (14%)	0.840
Neutral	83	18 (22%)	26 (31%)	30 (36%)	9 (11%)	
Disagree	40	9 (23%)	14 (35%)	12 (30%)	5 (13%)	
Pharmacists care about your health						
Agree	457	82 (18%)	156 (34%)	158 (35%)	61 (13%)	0.043
Neutral	76	10 (13%)	20 (26%)	35 (46%)	11 (15%)	
Disagree	66	16 (24%)	11 (17%)	31 (47%)	8 (12%)	
Pharmacists never mislead you about anything						
Agree	327	55 (17%)	125 (38%)	103 (32%)	44 (14%)	0.001
Neutral	142	23 (16%)	30 (21%)	69 (49%)	20 (14%)	
Disagree	130	30 (23%)	32 (25%)	52 (40%)	16 (12%)	
Pharmacists keep your sensitive medical information private						
Agree	380	60 (16%)	133 (35%)	127 (33%)	60 (16%)	0.009
Neutral	121	25 (21%)	30 (25%)	54 (45%)	12 (10%)	
Disagree	98	23 (24%)	24 (25%)	43 (44%)	8 (8%)	
You can tell pharmacists anything						
Agree	414	70 (17%)	146 (35%)	147 (36%)	51 (12%)	0.012
Neutral	78	15 (19%)	11 (14%)	36 (46%)	16 (21%)	
Disagree	107	23 (22%)	30 (28%)	41 (38%)	13 (12%)	

### Latent class groups according to preferences for HIV prevention services

**Figure 1:**
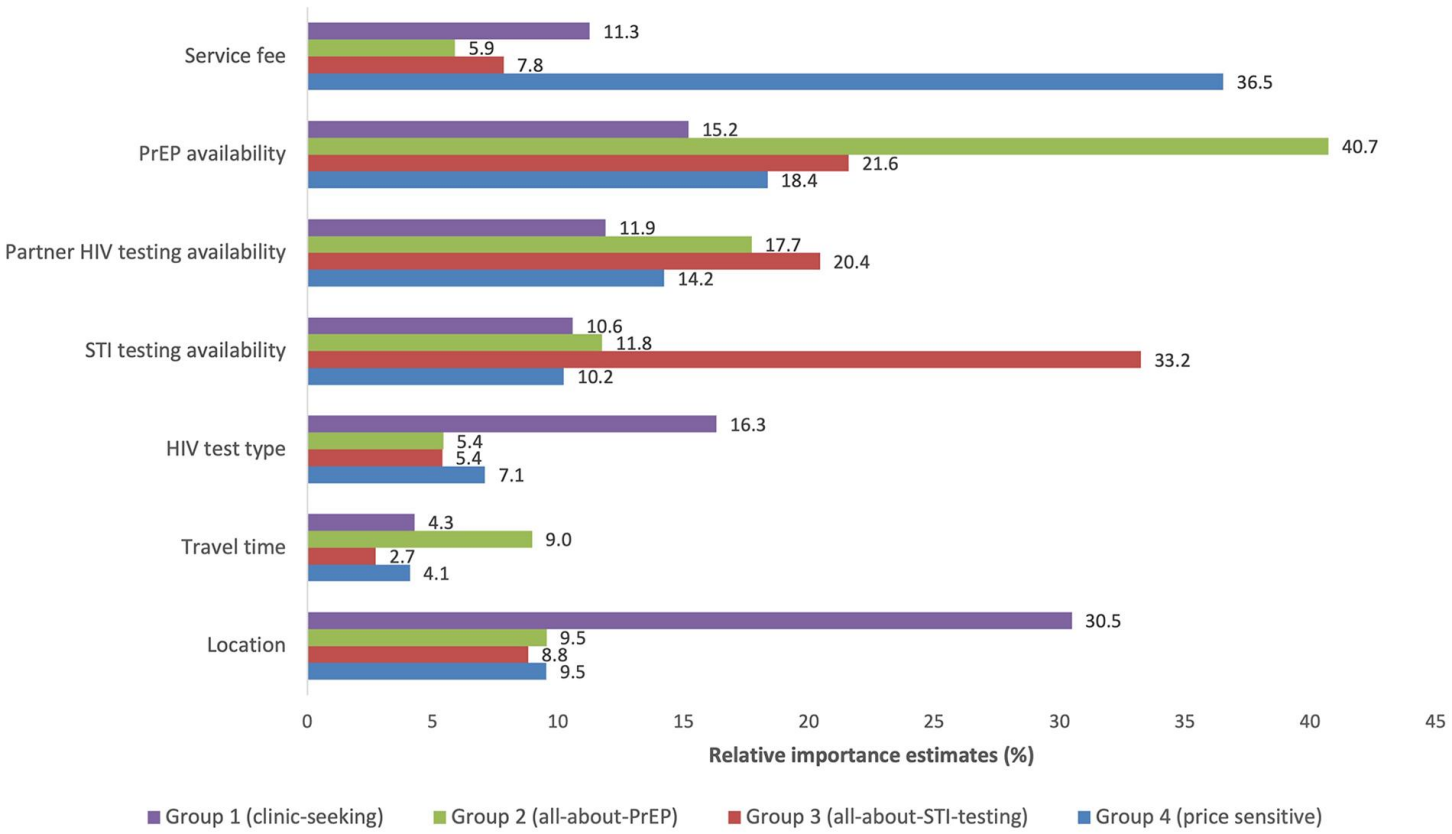
Relative importance of attributes by group.

The clinic-seeking group placed the highest relative importance on location (30.5%), compared with the other three groups (8.8% to 9.5%) (Figure 1). In this group, location was 1.9–7.1 times more important than the other attributes. Specifically, women in this group preferred receiving HIV prevention services at a clinic rather than at a pharmacy with a private room (0.27–0.02 = 0.25) or at a pharmacy without a private room [0.27–(−0.28) = 0.55] (Table 2). Women in this group also placed higher relative importance on HIV test type (16.3%) than the other groups (5.4% to 7.1%), preferring provider-administered testing to saliva or blood-based self-testing.

The all-about-PrEP group placed the highest relative importance on PrEP availability (40.7%), compared with the other groups (15.2% to 21.6%). In this group, PrEP availability was 2.3–7.5 times more important than the other attributes. Women in this group also placed higher relative importance on travel time (9%), compared with the other groups (2.7% to 4.3%), and preferred 15 min to 30 min [0.61–(−0.32) = 0.93] or 60 min [0.61–(−0.30) = 0.91]. Women in this group also placed lower relative importance on service fee (9%), compared with the other groups (7.7% to 36.5%). Women in this group preferred free services to services that cost 400 KES [0.31–(−0.30) = 0.61] or 1,000 KES [0.31–(−0.017) = 0.33; the 1,000 KES preference weight was not significant, *t*-ratio = −0.22]. Given that a larger negative preference weight was observed for the smaller fee compared with the larger fee, we ran an additional analysis with the service fee dummy-coded to explore whether this was an effects-coding artifact. Specifically, because effects coding constrains the three levels to sum to zero, a large positive preference weight (0.314 under free services) and a large negative preference weight (−0.297 under 400 KES) may force the remaining level (1,000 KES) toward zero. However, under dummy coding, with free as the reference, this same pattern persisted, with 400 KES having a more negative preference weight (−0.61; *t*-ratio = −3.91) than 1,000 KES (−0.329; *t* = −2.55).

The all-about-STI testing group placed the highest relative importance on STI testing availability (33.2%), compared with the other groups (10.2% to 11.8%). In this group, STI testing availability was 1.5–12.2 times more important than the other attributes. Women in this group slightly preferred going to a pharmacy with a private room to receive HIV prevention services over a clinic (0.21–0.14 = 0.07) and strongly preferred a pharmacy with a private room over one without [0.21–(−0.35) = 0.56]. Women in this group placed the lowest relative importance on travel time (2.7%), compared with other groups (4.1% to 9%).

Finally, the price-sensitive group placed the highest relative importance on the service fee (36.5%), compared with the other groups (5.9% to 11.5%). In this group, the service fee was 2–8.9 times more important than the other attributes. Women in this group preferred free services to those costing 400 KES (1.11–0.020 = 1.09) or 1,000 KES [1.11–(−1.13) = 2.24]. Women in this group also preferred receiving HIV prevention services at a pharmacy without a private room to one with a private room [0.26–(−0.31) = 0.57].

### Participant characteristics and attitudes to pharmacy services by latent class

The latent class groups differed significantly by select sociodemographic characteristics and attitudes toward pharmacy providers (Table 3). In Siaya, the clinic-seeking group comprised a higher proportion of participants (25%) than in the overall study sample (18%). In contrast, in Homa Bay, the clinic-seeking group comprised a lower proportion of participants (11%); the largest proportions were in the all-about-PrEP group (39% vs. 31% in the overall sample) and the all-about-STI testing group (39% vs. 37%). Among participants who disagreed that pharmacists care about their health, never mislead them, keep their sensitive medical information private, and that they can tell pharmacists anything, a higher proportion were in the clinic-seeking group and a lower proportion were in the all-about-PrEP group than in the overall sample. Among participants with at least some higher education, a smaller proportion were in the price-sensitive group (6%) than in the overall sample (13%).

## Discussion

We conducted a latent class analysis to determine whether subgroups of women with different preferences for receiving HIV prevention services exist. We identified four distinct groups, each with preferences primarily driven by one attribute: STI testing availability, PrEP availability, location, and service fee (listed in order of group size). Our findings largely confirm results from our previous analysis, which identified the most influential attributes on average across the study population, in order of importance: PrEP availability, STI testing availability, partner HIV testing availability, service fee, location, HIV test type, and travel time (8). However, the latent class analysis offers additional insights beyond average preferences, most notably the identification of groups that prioritize service location and cost.

Our findings have several implications for service design. First, because the largest groups in our sample prioritized STI testing and PrEP availability, expanding access to these interventions may have the greatest overall impact on service uptake. Notably, the all-about-STI testing group was the only one that preferred receiving HIV prevention services in a pharmacy with a private room rather than in a clinic. This suggests an opportunity to deliver more comprehensive HIV prevention packages in pharmacy settings, extending beyond traditional PrEP-focused models (5). Ongoing evaluations of pharmacy-based PrEP models in Kenya have reported a high prevalence of Chlamydia trachomatis (20%) among adolescent girls and young women who access contraception through pharmacies, suggesting the importance of including STI testing at these service delivery points (5). However, further research is needed to determine the extent to which private rooms are available across pharmacies and how availability varies across study counties. Notably, this group showed a small but statistically distinct preference for longer travel times over shorter ones, which may support prior studies suggesting that clients might be willing to pay more or travel farther for privacy reasons or to avoid stigma concerns, and aligns with this group's preference for pharmacies with private rooms over clinics or pharmacies without private rooms (17, 18).

Second, this study identified a subgroup, the “clinic-seeking group,” comprising approximately 18% of women, for whom clinic-based service delivery was strongly preferred over pharmacy-based options. While pharmacy-based models may expand access for many, these findings suggest they may be less acceptable for this subset of women. Our findings are consistent with other studies in the African region, which have demonstrated heterogeneity in preferences for the locations where HIV prevention services are delivered, with some groups preferring clinic- vs. pharmacy-delivered services (19, 20). Therefore, care must be taken to avoid leaving this group behind and to ensure targeted service improvement initiatives in clinic settings. This would be especially true in Siaya, which accounted for a larger proportion of the clinic-seeking group. That said, given that Homa Bay and Kisumu Counties had lower proportions of clinic-seeking participants, these counties could be optimal starting points for pharmacy-based programs.

Third, at the study's service fee threshold (a maximum of 1,000 KES), price-sensitive individuals made up a much smaller proportion of participants. However, further research is needed to evaluate whether women are willing to pay more than the amount tested in the study, and the thresholds at which cost is likely to be a major driver of uptake in this study population. Rather than broad service fee reductions, flexible cost-sharing models that include low-cost or free service options can help address the needs of price-sensitive individuals. Notably, this study found that price-sensitive individuals preferred pharmacies without a private room to those with one, which may be attributed to individuals perceiving pharmacies with a private room as more expensive. However, further qualitative research is needed to evaluate whether this is a genuine preference and whether private rooms affect women's perception of cost.

We acknowledge that the latent classes identified reflect how women chose the services presented to them in the DCE and may be limited by hypothetical bias, as what women choose might not necessarily reflect what they do in practice. Further, their contextual experiences—having lived in high HIV-burden counties in Kenya, participated in prior PrEP studies, and having general awareness of HIV prevention interventions due to community engagement in these counties—might also have affected their preferences and the resulting classes, and may be unique to the study population. However, not all women in our study had direct experience with PrEP access or use, and preference research benefits from including participants with varying levels of exposure to the services being evaluated. The salience of two-level attributes (availability vs. non-availability of HIV prevention services) may also have influenced the types of choices participants made, with service availability a critical factor in their choices. Further research is needed to determine how these services should be operationalized in pharmacy settings. The BIC favored the 3-class solution over the 4-class solution by a modest margin (change in BIC = 7.24); we therefore interpret findings specific to the smallest (clinic-seeking) class with appropriate caution. Finally, we did not collect current pregnancy status during the survey administration; instead, we asked participants to imagine a hypothetical pregnancy when making their choices. Therefore, we were unable to assess whether current pregnancy status influenced women's preferences for service options.

## Conclusions

In this study population, we identified four groups based on preferences for service location, PrEP availability, STI testing availability, and service fees. Heterogeneity in preferences suggests several design implications for delivering pharmacy-based HIV prevention services to pregnant women, including prioritizing STI testing and PrEP implementation, ensuring targeted improvements to clinic-based services for women who value this location, and offering flexible pricing to accommodate price-sensitive individuals.

## Data Availability

The datasets presented in this article are not readily available because The data supporting this study's findings are available from the corresponding author upon reasonable request. Requests to access the datasets should be directed to mugambi@uw.edu.
